# Stratification caused by sand extraction pits in coastal seas

**DOI:** 10.1038/s41598-026-48662-5

**Published:** 2026-05-25

**Authors:** Mohammad Daliri, Johan van der Molen

**Affiliations:** Department of Coastal Systems, Royal Netherlands Institute for Sea Research (NIOZ), 1790 AB Den Burg, Texel, The Netherlands

**Keywords:** Sand extraction, Stratification, Tidal mixing, Hydrodynamic modelling, Simpson–Hunter parameter, North Sea, Environmental sciences, Ocean sciences

## Abstract

As demand for marine sand increases across the globe, large-scale seabed excavation in shallow shelf seas raises concerns about its impact on local hydrodynamics and ecosystem functioning. In the Netherlands maritime area of the North Sea, deep sandpits excavated for coastal protection, beach nourishment, and infrastructure projects may reduce tidal mixing and promote thermal stratification, with implications for e.g. oxygen dynamics and biological productivity. This study evaluates how anthropogenic seabed deepening influences stratification onset, focusing on the role of sandpit geometry, including excavation depth and horizontal extent. We combine the energy-based Simpson–Hunter stratification model with a nested hydrodynamic modeling framework, employing GETM for large-scale circulation and Delft3D for high-resolution simulations using 16 sandpit scenarios. We introduce the concept of a critical sandpit depth, defined as the threshold depth beyond which thermal stratification may emerge in summer due to local deepening and associated reductions in tidal current speed. Results from the numerical model confirm that stratification tends to emerge when excavation depth exceeds the local critical threshold derived from the Simpson–Hunter criterion, with deeper and wider pits more likely to trigger stable vertical temperature gradients. In narrower pits, however, stratification is suppressed, likely due to insufficient water residence time relative to the local tidal excursion length. These findings provide a predictive tool for assessing the hydrographic consequences of sand extraction, supporting environmental planning under the Marine Strategy Framework Directive (MSFD). The approach is broadly applicable to other shelf systems for areas with limited salinity gradients, offering operational guidance for identifying excavation limits that prevent stratification and maintaining good environmental status (GES) in marine waters.

**Supplementary Information:**

The online version contains supplementary material available at 10.1038/s41598-026-48662-5.

## Introduction

Thermal stratification in shallow shelf seas is a fundamental process that influences oxygen availability, nutrient cycling, and biological productivity^1–3^. It results from the interplay between solar heating, which promotes surface-layer buoyancy, and tidal mixing, which acts to homogenize the water column^4^. In the North Sea, deep, low-energy areas like the Oyster Grounds stratify regularly^5^, while shallower, tidally energetic zones like the Dogger Bank and Southern Bight remain mixed^1,6^. The boundary between stratified and mixed waters is typically defined by a surface-to-bottom temperature difference of 0.5 °C^7^ and is well predicted by the Simpson–Hunter parameter, a dimensionless ratio comparing water depth to tidal current strength^4^.

While natural stratification patterns have been extensively studied in the North Sea and other shelf systems^8–11^, the localized impacts of anthropogenic seabed alteration such as sandpit excavation on stratification dynamics remains poorly understood. Excavated pits increase local water depth and often reduce tidal current velocity^12,13^, weakening turbulent energy and isolating the water column inside the pit from the surrounding flow, thereby creating conditions favorable to stratification^14^. This is particularly concerning because it may lead to substantial ecological changes, including altered oxygen levels and nutrient distributions, with cascading effects on benthic communities and pelagic food webs^3,15–17^.

The urgency of this issue is amplified by the accelerating demand for marine sand, particularly near the Dutch coast. Here, sand is mostly extracted for coastal maintenance and protection, and infrastructure and construction projects. The Netherlands is the largest extractor of marine sand among North Sea nations, removing over 25 million cubic meters annually^18^. A large portion of this sand, however, is used for coastal nourishment projects and therefore remains within the active coastal sediment system rather than being permanently removed. Globally, sand is the second most extracted resource after water, surpassing biomass and fossil fuels^19–21^, with expected annual demand reaching 60 billion tons by 2030^22–24^. Despite this scale, sand extraction remains one of the least regulated sectors globally^25^.

**Fig. 1:**
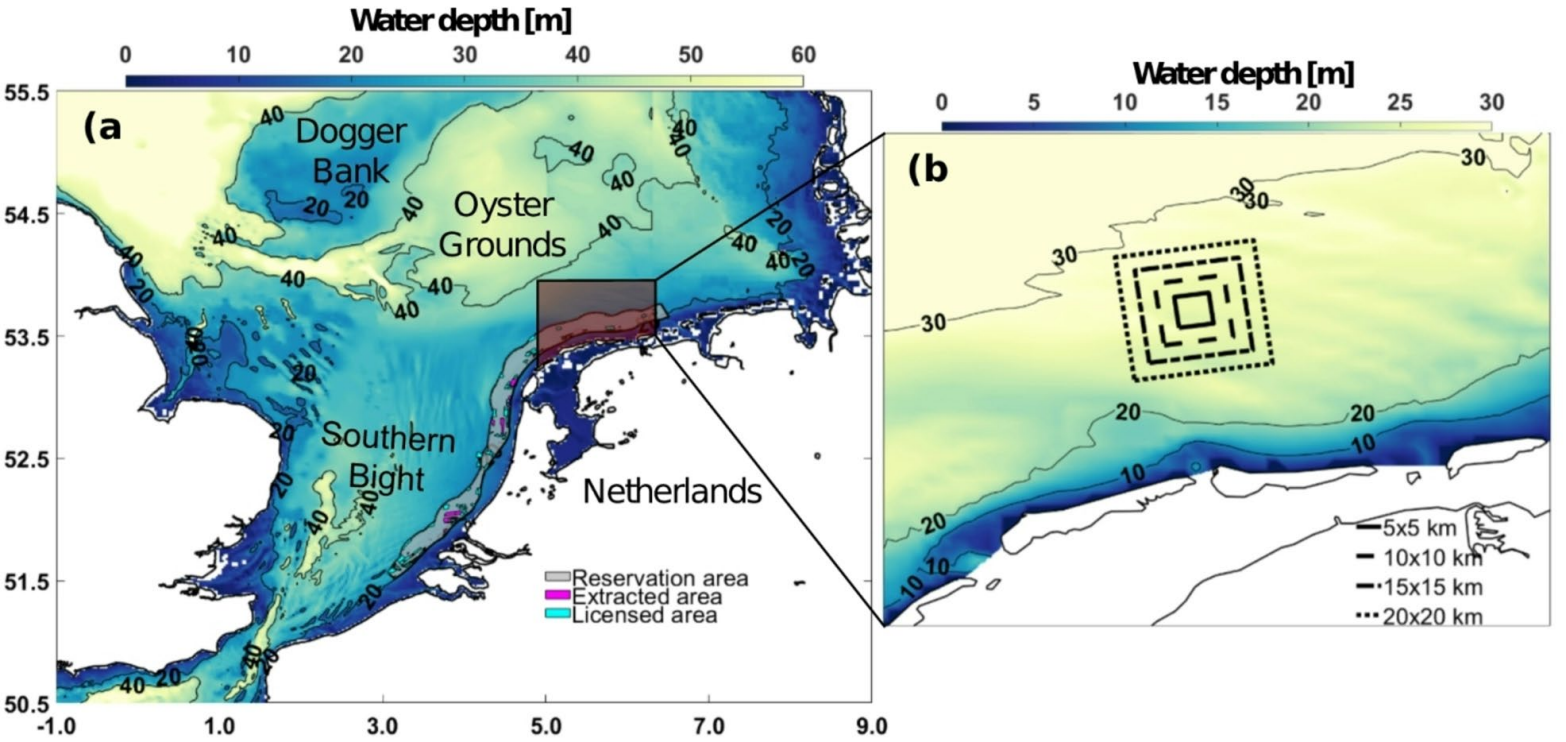
Study area and numerical model setup. (**a**) Bathymetry of the southern North Sea showing sand extraction zones defined under the Rijkswaterstaat extraction strategy: grey indicates reserved areas, magenta previously extracted areas, and cyan currently licensed areas (source: Rijkswaterstaat, https://www.noordzeeloket.nl/functies-gebruik/artikel-baseline/). (**b**) Model domain and sandpit configurations used in the simulations, comprising 16 square sandpit scenarios with widths of 5, 10, 15, and 20 km and excavation depths of 5, 10, 15, and 20 m. Maps generated using MATLAB R2023a (https://www.mathworks.com).

To manage domestic extraction, the Dutch national government (Rijksoverheid), has designated over 5000 km^2^ of offshore zones for sand mining, situated between the 12-nautical-mile boundary and the NAP (Normaal Amsterdams Peil, the Dutch national vertical reference level, roughly equivalent to local mean sea level) − 20 m depth contour. These zones are implemented and managed by Rijkswaterstaat, the Dutch authority responsible for water and coastal management. Within these areas, extractions deeper than 2 m are permitted, provided they occur at least 2 km seaward from the active − 20 m isobath (Fig. 1a). While spatial planning offers regulatory control, concerns persist about the environmental consequences of large-scale extraction. These include physical disturbance of the seabed and benthic habitats^26–28^, as well as long-term reductions in biodiversity and ecosystem functioning^24,29–31^. However, less attention has been given to how such excavations might alter the local hydrography and drive new patterns of water column stratification.

This study addresses that gap by quantifying how basic sandpit geometry, specifically excavation depth and horizontal extent, influence the onset of thermal stratification in the Dutch North Sea. We introduce the concept of a critical sandpit depth, defined as the threshold beyond which stratification is likely to emerge under local hydrodynamic flow conditions. To estimate this, we apply the energy-based Simpson–Hunter criterion that only considers tidal flow and depth, and then compared with via high-resolution numerical simulations using GETM for basin-scale circulation and Delft3D for local-scale processes, applied to 16 sandpit configurations of varying depth and size for a location with limited salinity gradients. Our findings provide a predictive and physically grounded framework to identify excavation thresholds that prevent stratification. This directly supports Descriptor 7 of the Marine Strategy Framework Directive (MSFD), which seeks to prevent permanent hydrographical alterations that negatively impact marine ecosystems^32^. The framework also may contribute to management and stakeholder education when integrated into emerging platforms such as the Digital Twin of the North Sea^33^ and the Maritime Spatial Planning (MSP) Challenge Simulation Platform^34^. Incorporating stratification thresholds into these platforms enables stakeholders to explore the effects of excavation depth and placement, enhancing their understanding and supporting informed decision-making.

While focused on the Dutch context, the methodology and insights are transferable to other shelf systems experiencing growing pressure from sand extraction. By linking excavation design to stratification risk, this work contributes to environmentally responsible seabed management and supports the achievement of Good Environmental Status (GES) in marine waters.

## Methodology

### Simpson–Hunter model

A simple yet powerful theoretical framework to determine whether a region in shelf seas will be thermally stratified was developed by Simpson and Hunter^4^. It is based on the balance between two competing forces: buoyancy input from surface heating, which creates a lighter surface layer and promotes stratification, and turbulent energy generated by tidal currents, which mixes the water column and acts to erode or prevent stratification. This balance is described using the concept of potential energy anomaly $\:\phi\:$, which represents how much energy is needed to mix the water column fully. Stratification forms when surface heating increases $\:\phi\:$ faster than tides can stir it away. To assess this balance, Simpson and Hunter introduced a non-dimensional ratio based on water depth $\:h\:$and depth-averaged tidal current speed, typically the $\:{M}_{2}$ component $\:{u}_{M2}$. Assuming that surface heating is roughly uniform and that mixing efficiency is constant, the ratio captures the interplay between buoyancy and mixing. Deeper water favors stratification, while stronger tidal currents favor mixing. For ease of comparison across different areas, this ratio is often expressed as the base-10 logarithmic value called the Simpson–Hunter parameter:

1 $$\:\begin{array}{c}SH={log}_{10}\left(h/{u}_{M2}^{3}\right)\end{array}$$

This parameter helps identify areas that are likely to stratify. Higher *\:SH* values suggest stratified conditions, while lower values indicate regions that remain mixed. The transition between these two regimes forms tidal mixing fronts, which often align with a characteristic *\:SH* value. For the northwest European shelf, previous studies starting from^4^ have reported a threshold around 2.7^35^, beyond which stratified conditions typically develop in summer. Across the southern North Sea, the eastern English Channel, and much of the Irish Sea, *\:SH* values are generally below this threshold, indicating well-mixed conditions.

As such, *\:SH* provides a convenient way to map and compare stratification potential across different parts of the shelf sea. It has also been widely applied in theoretical and numerical studies to assess stratification variability and helps identify areas at risk of oxygen depletion or shifts in nutrient dynamics due to changes in mixing^36,37^. It is worth noting that throughout the stratified season, the tidal mixing front in the North Sea remains remarkably stable, making it a reliable feature for studying hydrodynamic responses to local changes. Semi-diurnal tidal currents cause daily shifts in the front’s position, usually between 5 and 15 km, but these movements are fully reversible and do not alter the overall stratification pattern^38-40^. Over longer periods, the spring–neap tidal cycle causes slower and smaller adjustments. Although theory suggests that stronger mixing during spring tides could move the front by 10–20 km, observations show that it usually shifts only 2–4 km^41,42^. This smaller response occurs because (i) stratification builds up during neap tides and stores buoyancy, and (ii) once formed, it reduces the mixing efficiency of turbulence^43,44^. As a result, part of the spring-tide energy is used to erode existing stratification rather than to move the front. These processes have been confirmed by satellite observations and modeling^7,40^ and observed in other shelf seas such as the Bungo Channel and Georges Bank^39,45,46^. Consequently, the frontal boundary stays closely aligned with the *\:SH* ≈ 2.7 contour, confirming the robustness of this threshold value.

### Calculation of critical sandpit depth

To estimate the maximum allowable excavation depth before stratification occurs, we adapted the Simpson–Hunter formulation using a local velocity correction. The approach assumes that tidal velocities within a pit are reduced due to continuity, which in turn increases the local *\:SH* value. By quantifying this reduction, we derive an expression for the critical pit depth under site-specific tidal conditions.

Assuming mass conservation, negligible lateral changes in velocity, and a uniform cross-sectional shape, the reduced local velocity within the pit, $\:{u}_{p}$, can be estimated using a continuity correction:

2 $$\:\begin{array}{c}{u}_{p}=u\frac{h}{{h}_{p}}\end{array}$$

Where *\:u* and *\:h* are local velocity and water depth outside the sand pit respectively. $\:{h}_{p}=h+\:{d}_{p}$ is the water depth over the sand pit where $\:{d}_{p}$ is the sand pit depth relative to the original seabed.

To determine the maximum pit depth that still avoids stratification, we impose a critical Simpson–Hunter value, $\:{SH}_{crit}$, above which stratification is expected to occur. The continuity-corrected velocity $\:{u}_{p}$ derived in Eq. (2) is substituted into the Simpson–Hunter formulation to account for the reduction in tidal velocity caused by local deepening. This corresponds to a critical total water depth $\:{h}_{p}^{crit}$, and we define the critical excavation depth as:

3 $$\:\begin{array}{c}{d}_{p}^{crit}=\:{h}_{p}^{crit}-h\end{array}$$

Substituting the continuity-corrected velocity into the *\:SH* expression,

4 $$\:\begin{array}{c}{SH}_{crit}={log}_{10}\left[\frac{{h}_{p}^{crit}}{{\left({u}_{M2}h/{h}_{p}^{crit}\right)}^{3}}\right]\end{array}$$

And solving for $\:{d}_{p}^{crit}$, we obtain:

5 $$\:\begin{array}{c}{d}_{p}^{crit}={10}^{{SH}_{crit}}\:{\left[{u}_{M2}\left(\frac{1}{1+{d}_{p}^{crit}/h}\right)\right]}^{3}-h\end{array}$$

This defines the maximum allowable pit depth under given tidal conditions that maintains the local *\:SH* below the stratification threshold. As Eq. (5) is implicit in $\:{d}_{p}^{crit}$ its value is determined through an iterative root-finding method that solves the nonlinear equation.

### Numerical model

To investigate the effects of sandpit excavation on hydrodynamics and thermal stratification in more detail, a one-way nested numerical modeling approach was employed. The General Estuarine Transport Model (GETM) was used as the outer model to simulate large-scale hydrodynamic processes, while the Delft3D model was applied as the inner model to resolve high-resolution local dynamics at the sandpit location. GETM is a three-dimensional, baroclinic hydrodynamic model that solves the shallow-water, heat balance, and density Equations^47,48^. GETM uses the General Ocean Turbulence Model (GOTM)^49^ to resolve vertical processes, including turbulence closure schemes, and operates on a staggered Arakawa C-grid with general vertical coordinates. This enables it to simulate key physical processes such as sea surface elevation, currents, temperature, salinity, and drying and flooding of land cells. The regional GETM setup used here was originally developed for the north-west European shelf^50^. For the inner domain, Delft3D was selected because it allows efficient testing of idealized sandpit geometries and controlled scenario analysis, while also providing a more complete treatment of solar radiation in the heat-flux calculation, which is important for stratification development. In this study, GETM was configured on a spherical grid covering the North Sea region (46.4° N–63° N latitude and 17.25° W–13° E longitude) with a horizontal resolution of 0.02° longitude and 0.05° latitude (~ 5 km) and 25 non-equidistant vertical layers^50,51^. Bathymetric data were sourced from the North West European Shelf Operational Oceanographic System (NOOS). Tidal forcing was applied using TOPEX/POSEIDON satellite altimetry^52^, and atmospheric forcing was provided by ECMWF ERA5 and operational reanalysis datasets^53^. River runoff inputs were implemented using the ICG-EMO riverine database^54^, while depth-resolved temperature and salinity boundary conditions were extracted from ECMWF-ORAS4 data^55,56^. The accuracy of GETM in reproducing tidal dynamics has been demonstrated by^50^, who validated the model’s $\:{M}_{2}$ tidal component against observational data in the North Sea, showing strong agreement for both tidal elevations and current velocities.

Delft3D is a three-dimensional hydrodynamic model capable of simulating flow, sediment transport, and morphological changes in coastal environments^57^. The model reliably resolves tidal currents, turbulence, and density-driven flows and has been widely used to assess seabed alterations such as dredging and sand extraction^58,59^. The grid focuses on a smaller region in the Dutch North Sea, situated between the Vlieland and Schiermonnikoog islands, spanning 52.0° N–54.5° N latitude and 3.0° E–8.0° E longitude. The bathymetry in this area ranges from shallow coastal waters (< 5 m) to offshore regions reaching 40 m (provided by NOOS). A horizontal resolution of 200 m was used to capture small-scale flow patterns and seabed topography variations, particularly over the hypothetical sandpit. The applied bathymetry includes larger seabed bedforms present in the study area, allowing part of their influence on near-bed turbulence to be represented directly. Smaller unresolved roughness effects are accounted for through bottom roughness parameterization, using a bed roughness length that includes both grain-related and ripple-induced roughness. Because the same surrounding bathymetry is used in all scenarios, relative differences between simulations remain primarily controlled by sandpit geometry. For the vertical discretization, a Z-grid model with 25 fixed layers was implemented to accurately resolve thermal stratification. The Z-grid configuration maintains horizontal layers, minimizing numerical errors over steep slopes, such as the sandpit, and preserves sharp thermal gradients and pycnoclines while reducing artificial numerical mixing^60^.

Figure 1b shows the model setup and the full set of sandpit scenarios tested in this study. We performed simulations across all combinations of four sandpit sizes [5, 10, 15, 20] km and four depths [5, 10, 15, 20] m, resulting in a total of 16 square pit configurations. Each scenario was simulated under identical hydrodynamic and atmospheric forcing conditions to isolate the effects of geometry, over a representative summer period spanning June–July 2019.

The nesting process involved linearly interpolating boundary data from GETM to the Delft3D grid. For each open boundary, water levels, temperature, and salinity fields from GETM were interpolated across multiple sections to ensure a smooth and accurate transfer of conditions into the Delft3D domain. Atmospheric forcing was applied using ECMWF ERA-5 data, while the heat flux was calculated based on the formulations of^61,62^. These formulations, calibrated specifically for the North Sea, compute solar radiation dynamically as a function of cloudiness, air temperature, and relative humidity. The solar radiation flux accounts for the Earth’s position and local time, providing a precise representation of surface heating processes.

## Model validation

To evaluate the accuracy of the numerical modeling framework, we conducted a two-step validation focused on water temperature, the primary variable governing thermal stratification. Field observations of water temperature were obtained from the ICES (International Council for the Exploration of the Sea) Ocean hydrochemistry database (ICES DataPortal), covering the period from 2017 to 2019. However, these observations are highly sparse and irregular in both space and time. They were collected at varying depths, from different locations, and on non-consecutive days. For the specific simulation period of interest (June–July 2019), no measurements exist for June within the nested domain, and only one observation station was available in July (at 6.30°E, 53.88°N), located near the eastern boundary of the Delft3D domain. At this station, the observed temperature difference between surface and bottom was 0.055 °C, while the model produced 0.10 °C at the same location. Absolute temperatures were also similar, with observed values of 16.575 °C at the surface and 16.52 °C near the bottom, compared with modelled values of approximately 16.93 °C and 16.83 °C, respectively. Supplementary Fig. S1 shows the spatial distribution of all available ICES observations from 2017 to 2019 over a wider area, aggregated across depths and time.

**Fig. 2:**
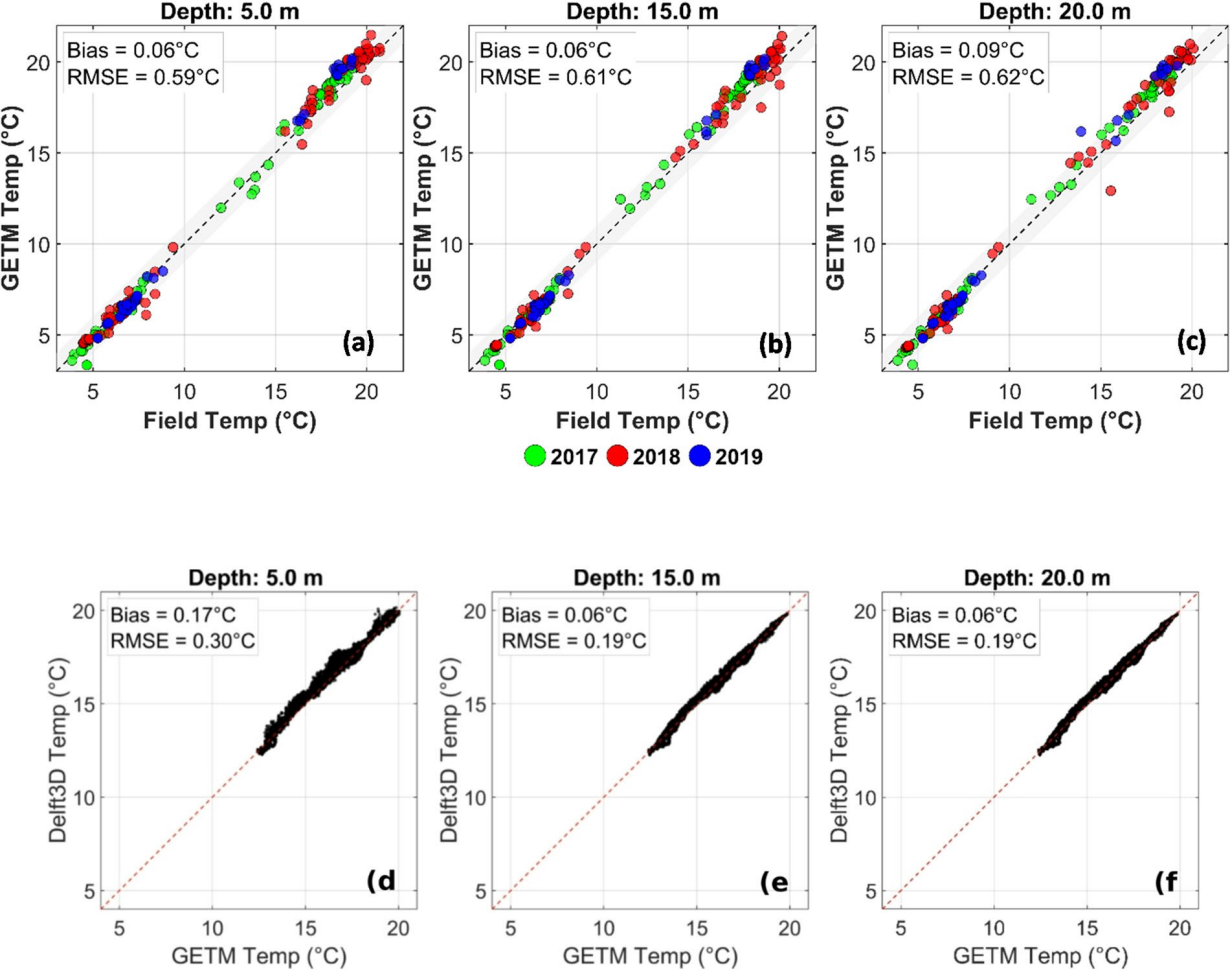
Validation of modelled temperature. Panels (**a**) to (**c**): Comparison between GETM-simulated temperatures and ICES observations at 5, 15, and 20 m depths using all available ICES data from 2017 to 2019. Each point represents a matched pair at the same time, location, and depth. Panels (**d**) to (**f**): Inter-model comparison between Delft3D and GETM at the same depths, using 3-hourly outputs from both models over June–July 2019, restricted to the area corresponding to the largest sandpit scenario (with flat seabed). The 1:1 dashed line indicates perfect agreement.

A direct validation of the Delft3D model within the nested domain was limited by the lack of observations matching the simulation period and location. To address this, we first validated the outer GETM model, which spans a wider area and a longer period of time with more consistent observational coverage, using the ICES dataset. Then, we performed an indirect validation of the Delft3D model by comparing its outputs to the GETM results. Figure 2 summarizes the outcomes of both validation steps. Figure 2a–c show scatterplots comparing GETM-simulated temperatures against ICES field observations at three representative depths: 5.0 m, 15.0 m, and 20.0 m. Each point represents a single field measurement matched to the corresponding GETM output at the same time, location, and depth, using all available ICES data from 2017 to 2019. Data are colored by year: green for 2017, red for 2018, and blue for 2019. The agreement is consistently strong across depths, with biases of 0.06–0.09 °C and RMSE values below 0.65 °C. The alignment of points along the 1:1 reference line demonstrates that GETM effectively captures the observed thermal structure throughout the region and over multiple years.

Figure 2d–f show the second step of the validation, which involved comparing Delft3D and GETM outputs at 5.0 m, 15.0 m, and 20.0 m depths during the simulation period from June to July 2019. For this comparison, the Delft3D nested model was configured with the same horizontal resolution as the GETM model, using an unmodified seabed without a sand excavation pit. This setup was chosen to ensure that the comparison was not affected by discrepancies arising from grid resolution, local topographic changes, or processes happening at spatial scales smaller than the GETM model resolution. This allowed us to isolate the performance of Delft3D and confirm that it reproduced the temperature structure generated by GETM under matching conditions within the target area. The analysis was restricted to the set of grid cells that spatially corresponds to the area where the largest sandpit would later be located, allowing a focused evaluation of model consistency over the target region. Temperature values from both models were extracted at every grid point within that area and every 3 h, yielding thousands of matched data pairs per depth level. The scatter plots show a tight distribution around the 1:1 line, with minimal bias. At 5.0 m depth, Delft3D shows a slightly warm bias of 0.17 °C and an RMSE of 0.30 °C. At 15.0 m and 20.0 m, the bias is even lower (0.06 °C), and RMSE drops to 0.19 °C. These small deviations are well within acceptable limits for hydrodynamic modeling, especially considering the differences in vertical mixing schemes and grid structure between the two models. The consistent agreement across all depths demonstrates the reliability of the nested modeling approach in capturing temperature gradients relevant to stratification. Moreover, this two-step validation confirms that the model setup is suitable for the stratification analysis in this study.

## Results

### Large-scale thermal structure from GETM

To establish a physical baseline for summer stratification in the Dutch North Sea and evaluate theoretical thresholds, we analyze large-scale temperature fields simulated by the GETM model. Supplementary Fig. S2 shows mean surface temperature, bottom temperature, and vertical temperature difference ($\:\:\varDelta\:T=$ $\:{T}_{surface}$ - $\:{T}_{bottom}$) averaged over June–July 2019. Surface waters (Supplementary Fig. S2a) are relatively uniform, reaching ~ 16 °C across most of the southern and central basin, with cooler patches (< 14 °C) in the northwest. Bottom temperatures (Supplementary Fig. S2b) exhibit a clear north-south gradient, remaining > 14 °C over the shallow southern shelf and Dogger Bank, and dropping below 10 °C in the deeper northern basin beyond 54.5°N, reflecting strong seasonal stratification. Supplementary Fig. S2c shows $\:\varDelta\:T$ as a measure of stratification strength; the color scale is limited to 0–1 °C to emphasize the 0.5 °C isotherm, commonly used to delineate the tidal mixing front^4,63^. This front provides a reference for assessing how local bathymetric changes, such as sandpit excavation, may influence stratification dynamics.

**Fig. 3:**
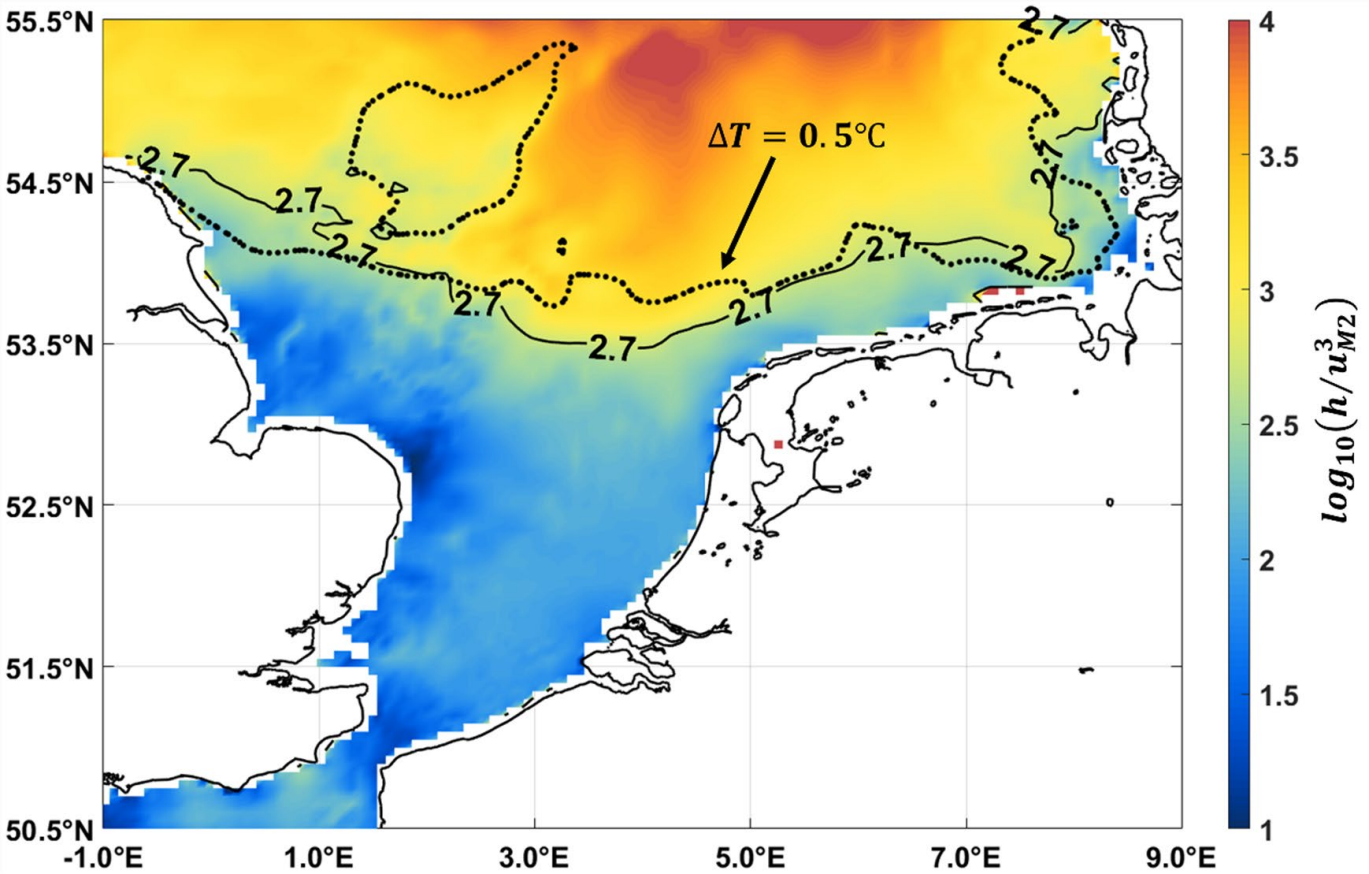
Spatial distribution of the Simpson–Hunter parameter $\:SH={log}_{10}\left(h/{u}_{M2}^{3}\right)$computed from local bathymetry and M₂ tidal current amplitude. The black contour shows the $\:\varDelta\:T=$0.5 °C mixing front from Supplementary Fig. S2c*.* Map generated using MATLAB R2023a (https://www.mathworks.com).

To further interpret the location of the thermal front, we compare it with the Simpson–Hunter parameter in Eq. (1). Figure 3 shows the spatial distribution of the Simpson–Hunter parameter (*\:SH*). Higher values (> 2.7) occur in deeper northern areas prone to stratification, while lower values (< 2.7) dominate the well-mixed southern shelf. The $\:\varDelta\:T=$ 0.5 °C contour from Supplementary Fig. S2c aligns with the $\:SH\approx\:$2.7 threshold, confirming that stratification emerges where tidal mixing is too weak to overcome buoyancy. The model output clearly defines the summer mixing front and provides a physically consistent reference state for examining how local seabed changes may alter stratification dynamics.

### Simpson–Hunter model results

**Fig. 4:**
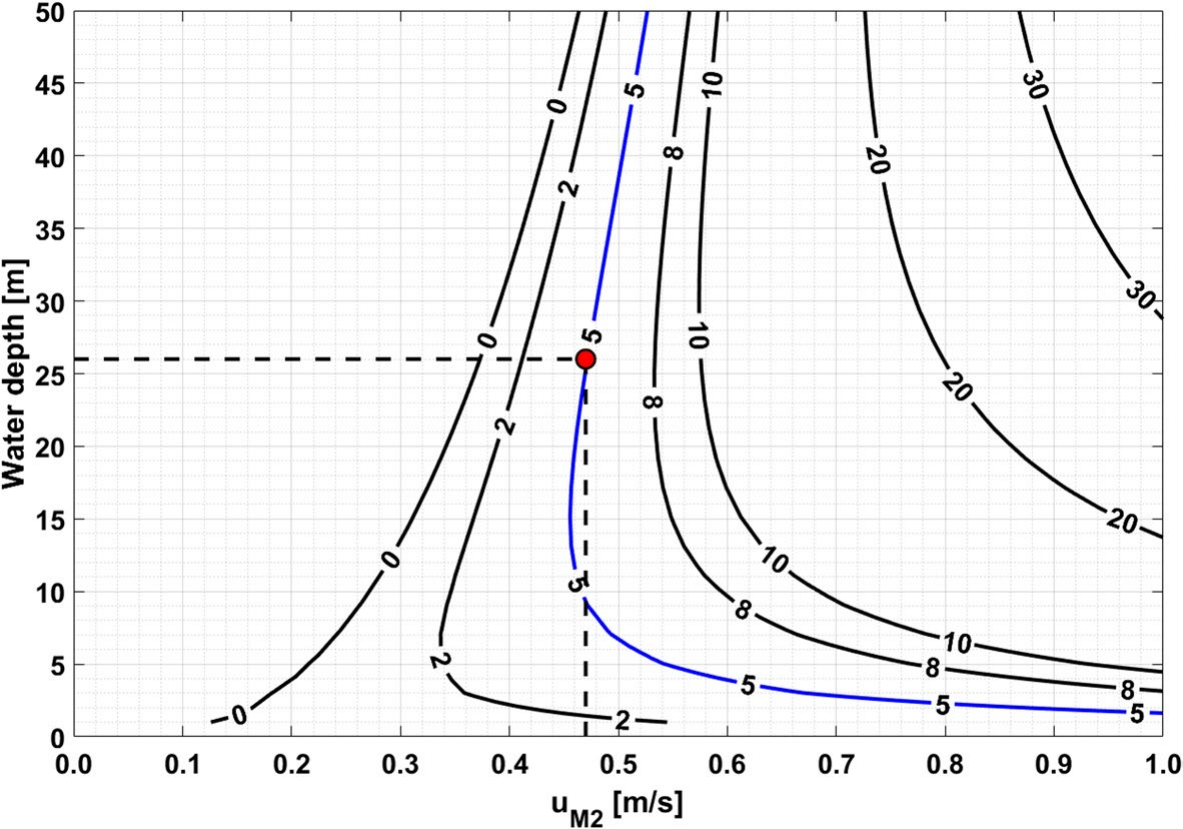
Maximum allowable sandpit depth accounting for velocity reduction due to bathymetric deepening according to Eq. (5), with an example showing a critical depth of 5 m for a water depth of 26 m and an M_2_ tidal velocity amplitude of 0.47 ms^−1^.

Building on Eq. (5), we compute the critical excavation depth $\:{d}_{crit\:}$ from the continuity-adjusted Simpson–Hunter expression using $\:{SH}_{crit}=2.7$ (Sect.  4.1). To generalize beyond a single site, we solve $\:{d}_{crit\:}$ over background depths *\:h*from 0 to 50 m and tidal velocity amplitudes $\:{u}_{M2}$ from 0 to 1 m/s. Figure 4 presents contours of $\:{d}_{crit\:}$, giving the maximum allowable pit depth for each depth-velocity pair. The pattern shows that the effect of ambient water depth changes with tidal velocity. At lower tidal velocities, deeper water needs less additional pit depth than shallow water to reach stratified conditions. This is because vertical mixing is relatively weak, so ambient depth becomes the main controlling factor, and deeper water is already closer to the stratification threshold. At higher tidal velocities, this behavior reverses. In that case, vertical mixing is much stronger and makes stratification harder to develop. As a result, ambient depth alone is no longer the main control, and the relative depth increase caused by the pit becomes more important. For the same excavation depth, the proportional change in water depth is larger in shallow water than in deeper water, so the pit has a stronger effect in shallow areas. This is why, under stronger tidal mixing, shallow water can require less pit depth than deeper water to reach the same stratification threshold. Because the formulation includes the reduction of tidal velocity over a deepened pit, these limits are more conservative than depth-only estimates that assume constant velocity.

**Fig. 5:**
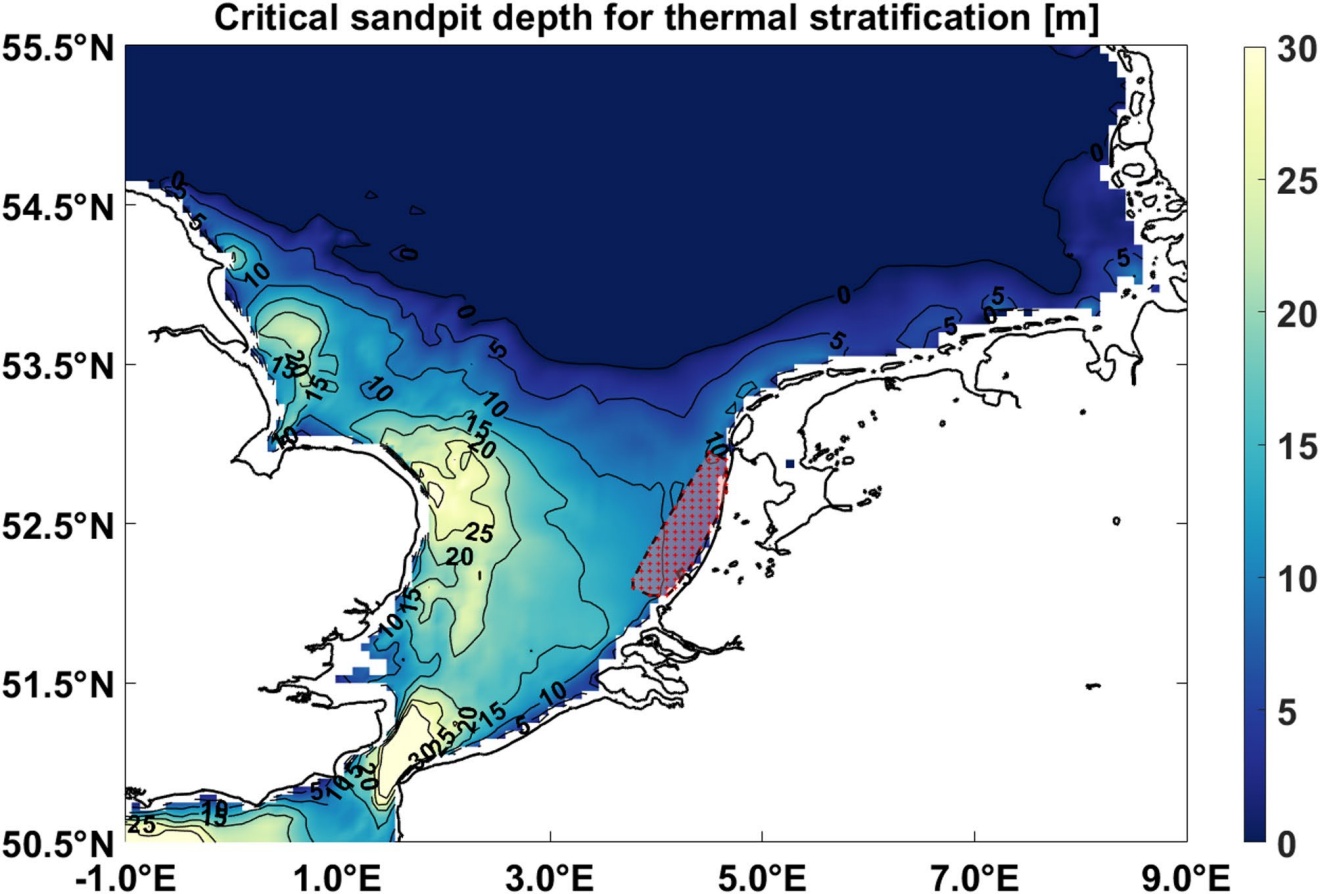
Spatial map of critical sandpit depths across the Dutch North Sea, showing the maximum excavation depth allowed before thermal stratification may occur under local tidal conditions. The Rhine ROFI that overlap parts of the sand extraction zone are masked (red hatching) to exclude salinity-driven effects. Map generated using MATLAB R2023a (https://www.mathworks.com).

Applying this refined approach across the Dutch North Sea using the M_2_ tidal velocity amplitudes from the GETM model, we derived a spatial map of critical sandpit depths (see Fig. 5). This map shows, for each grid cell, the maximum depth by which the seabed can be excavated without inducing stratification. Notably, the zero-depth contour in this map, indicating areas where stratification can occur without additional deepening, closely matches the *SH=* 2.7 contour from Fig. 3 and the $\varDelta\:T=$ 0.5 °C frontal boundary from Supplementary Fig. S2c. Regions with greater mixing potential (low SH values) allow for deeper excavation without risking stratification. It is important to note that this analysis considers only thermal stratification and does not account for salinity-driven density gradients. As a result, regions whose general hydrodynamics are influenced by salinity stratification, particularly the Rhine Region of Freshwater Influence (ROFI), were excluded from the map shown in Fig. 5. To exclude the influence of the Rhine Region of Freshwater Influence (ROFI) from the analysis, we masked the part of the southern Dutch coastal zone that is persistently modified by river-driven stratification. Observational studies indicate that the Rhine ROFI typically extends 20–40 km offshore and 100–120 km downstream^64,65^. Based on this footprint, we applied a ROFI mask covering 0–40 km offshore and 0–110 km alongshore from the Rhine mouth, isolating thermally controlled stratification patterns. These results emphasize that increases in depth in certain locations can trigger stratification by both increasing water column stability and reducing tidal energy. This is particularly relevant when compared with earlier studies such as^66^, which suggest that thermal layering in the North Sea typically occurs only when total water depth exceeds 40 m, consistent with the general depth of tidal fronts. While that benchmark holds for large-scale stratification patterns, it may overlook localized velocity reductions induced by excavation features such as sand pits. As demonstrated here, local deepening and flow reduction can lead to stratification onset at much shallower depths than previously assumed. The combination of Figs. 4 and 5 provides a physically grounded screening tool for assessing stratification risk associated with planned sand extraction activities.

### Delft3d results

Building on Sect.  4.2, we evaluate how well the predicted critical sandpit depths correspond to the onset of stratification ΔT > 0.5 °C when sandpit size is included in Delft3D hydrodynamic simulations. The analysis focuses on how combinations of pit depth and horizontal extent control stratification patterns under consistent forcing conditions. To isolate temperature-driven stratification, the model domain was located away from the Rhine Region of Freshwater Influence (Fig. 1a), minimizing salinity effects. The selected site has a natural water depth of ~ 26 m and a depth-averaged *\:M2* tidal velocity of 0.47 m s^−1^, yielding a predicted critical sandpit depth of ~ 5 m based on Figs. 4 and 5. Excavations deeper than this threshold are therefore expected to trigger thermal stratification.  

**Fig. 6:**
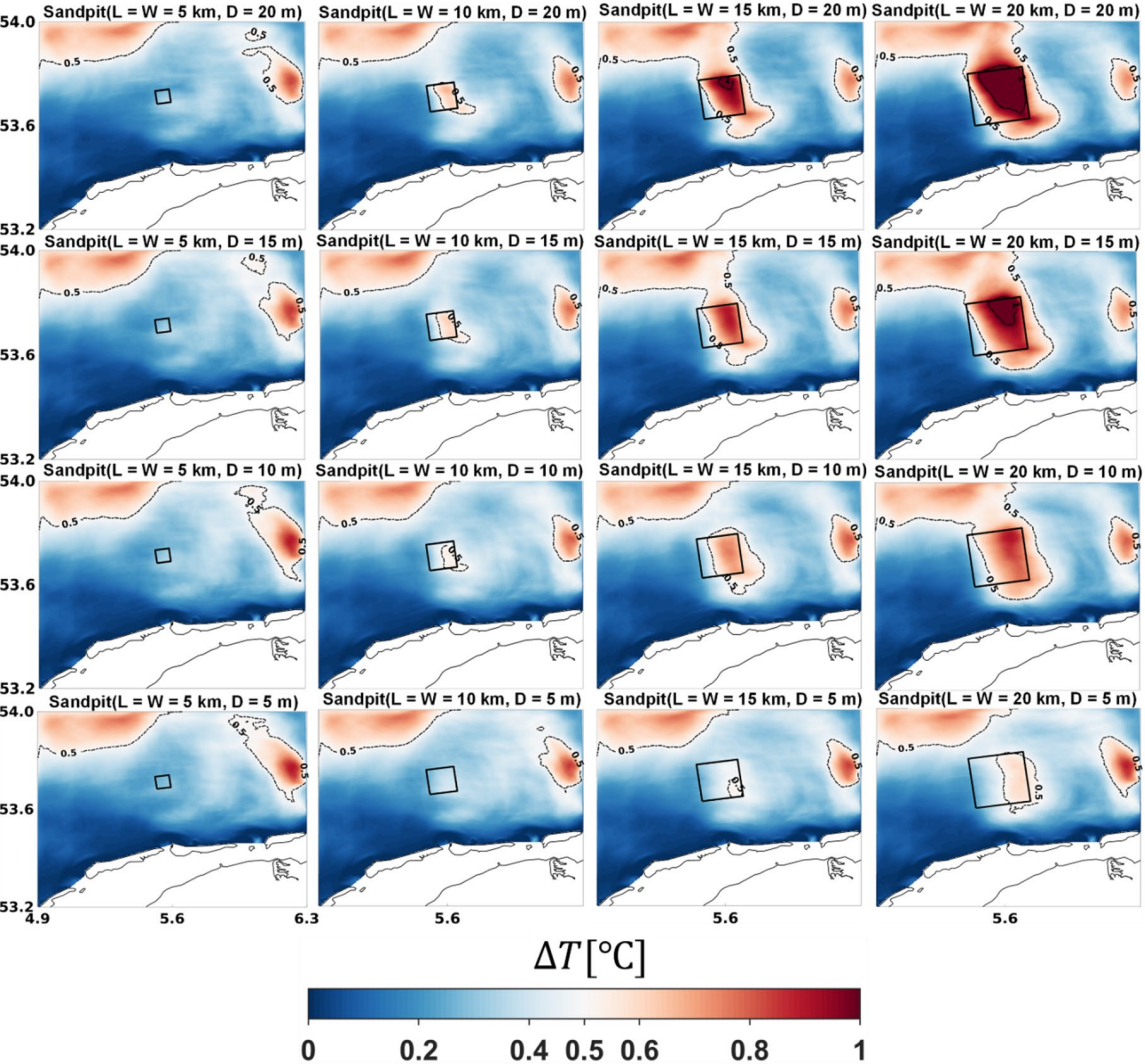
Spatial distribution of vertical temperature difference ($\:\varDelta\:T$) for all 16 sandpit scenarios, averaged over June–July 2019. Panel titles show pit length (L), width (W), and depth (D). Rows represent increasing excavation depths (D = 5–20 m, bottom to top), and columns represent increasing pit dimensions (L = W= 5–20 km, left to right). Maps generated using MATLAB R2023a (https://www.mathworks.com).

Figure 6 shows the spatial distribution of $\:\varDelta\:T$, averaged over June–July 2019, for all sandpit configurations. Rows represent excavation depths (5–20 m, bottom to top) and columns pit widths (5–20 km, left to right). The bottom row corresponds to the predicted 5 m critical depth, showing emerging stratification across varying pit widths. Here, stratification remains negligible in the 5 km and 10 km wide pits, but begins to emerge in the 15 km and 20 km cases. In the latter two cases, $\:\varDelta\:T$ only slightly exceeds the 0.5 °C threshold, indicating the initial onset of stratification. These results suggest that 5 m marks the depth at which the system begins to transition from mixed to stratified, provided that the excavation is wide enough to weaken local mixing. This supports the theoretical prediction and shows that the model can detect the earliest signs of stratification in conditions close to the threshold, consistent with the Simpson–Hunter framework.

As both excavation depth and width increase, stratified conditions expand spatially and intensify; the physical role of pit width is discussed further below. The most intense and widespread stratification appears in the largest, deepest pits (20 km × 20 m), where $\:\varDelta\:T$ exceeds the 0.5 °C threshold across most of the pit interior. Intermediate cases, such as the 15 km × 15 m pit, also exhibit notable but less extensive stratification. Across all sandpit depths, the 5 km width consistently results in mixed conditions, indicating that this horizontal scale is insufficient to trigger stratification even when combined with deeper excavations.

**Fig. 7:**
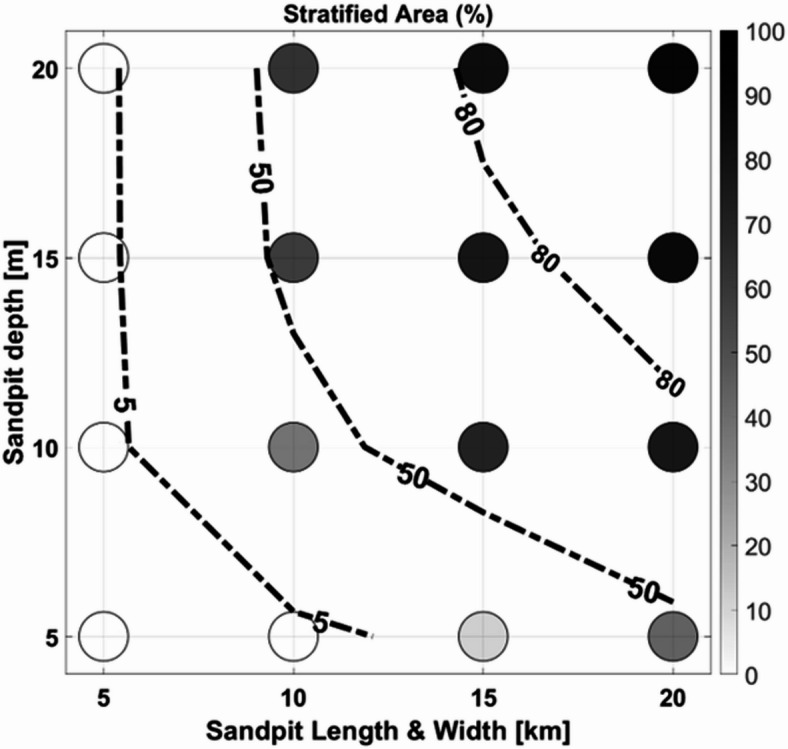
Stratified area percentage ($\:\varDelta\:T$ ≥ 0.5 °C) for each sandpit configuration.

This pattern is further quantified in Fig. 7, which shows the percentage of each sandpit area experiencing $\:\varDelta\:T$ ≥ 0.5 °C. Stratification covers more than 85% of the area in the largest/deepest configurations, while small, shallow pits remain nearly fully mixed. Notably, for the 10 km wide sandpit, no stratification occurs at a 5 m depth, but a 10 m deep excavation produces stratification over approximately 40% of the area. This suggests that the onset of stratification for the 10 km pit likely occurs slightly above 5 m, closely matching the predicted critical depth and demonstrating the sensitivity of stratification onset to fine-scale depth increments.

### Role of tidal excursion length

**Fig. 8:**
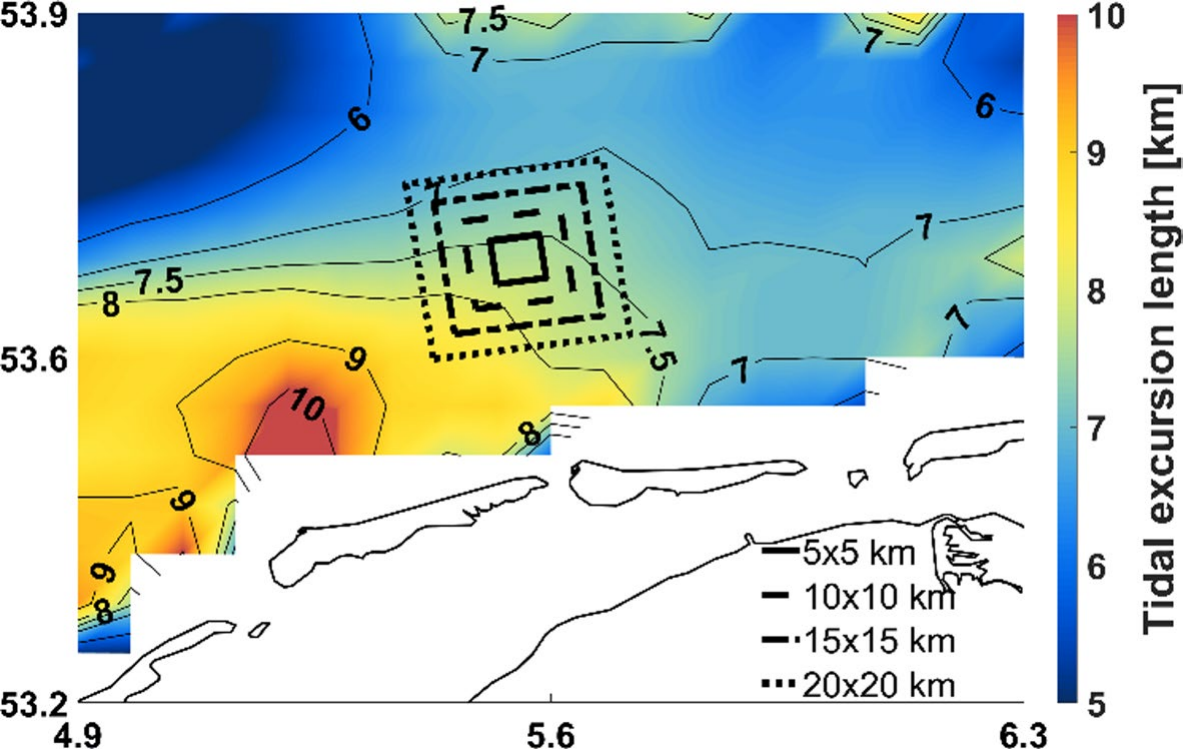
Spatial distribution of tidal excursion length [km], calculated as the average tidal displacement over June–July 2019 using depth-averaged velocities. Overlaid are the four sandpit widths considered in the study. Map generated using MATLAB R2023a (https://www.mathworks.com).

In addition to energy-based stratification thresholds, tidal excursion length may offer a complementary explanation for the absence of stratification in smaller sandpits. Tidal excursion, the horizontal displacement of a water parcel over a tidal cycle, governs the residence time of water within a sandpit. When a pit is smaller than the local excursion length, the water is rapidly flushed in and out, preventing the same body of water from remaining in the pit long enough to absorb sufficient solar radiation necessary for developing thermal stratification. In contrast, when the pit is larger than the excursion length, the same parcel of water tends to remain within the pit during the tidal cycle, allowing it to absorb heat and progressively stratify. Tidal excursion length was estimated by calculating the average horizontal displacement of depth-averaged tidal currents over the simulation period. The resulting map (Fig. 8) shows that excursion values in the vicinity of the sandpit site vary from about 7 km in the northeast part of the pit to 8.5 km in the southwest. Overlaid on the excursion field are the four pit widths tested in the simulations: 5, 10, 15, and 20 km. Interestingly, the spatial pattern of stratification in Fig. 6 aligns with this variation, as stratification tends to initiate in the northeastern portion of the pit where tidal excursion is smallest. Results show that pits narrower than the local excursion length, specifically those 5 km wide, do not stratify, regardless of depth. In contrast, stratification is consistently simulated in pits with widths of 15 km and 20 km, including at the critical 5 m depth. Intermediate-width pits, 10 km, only stratify when excavated beyond 5 m, indicating that the effective threshold for stratification onset lies near the tidal excursion length. These findings suggest that pit width must exceed the local tidal excursion in order for water to remain within the pit long enough to heat up and stratify. Otherwise, the continuous flushing of water inhibits the formation of stable thermal gradients. This supports the idea that tidal excursion may act as a limiting factor, even when energetic conditions for stratification are met based on pit depth. Overall, this emphasizes the need to jointly consider both local hydrodynamic conditions and the spatial characteristics of seabed modifications when evaluating the likelihood of stratification.

## Discussion

### Comparison with existing sand extraction cases in the North Sea

We compare modeled sandpit sizes and depths with southern North Sea case studies to identify the scales at which hydrodynamic and stratification effects may emerge under realistic coastal-management conditions and become detectable in temperature observations. Previous numerical studies considered pits from a few kilometers to > 100 km in length and ~ 2–20 m in depth^67–69^; although focused on tides, currents, and sediment transport, they illustrate the range of sandpit dimensions previously assessed in the region and provide useful geometric context for the present work.

Among North Sea sand-extraction sites, the Lowered Dumping Site (LDS) of the PUTMOR project is one of the best documented. Located 10–15 km west of Hoek van Holland, six pits of approximately 1300 × 500 m and 5–12 m depth were excavated at a water depth of ~ 23 m^66,70^. The main pit remained open for nearly 10 months before being refilled with dredged material from the Port of Rotterdam, allowing for extended monitoring of hydrodynamics, oxygen conditions, sediment transport, and morphology; however, no thermal stratification was observed. Applying the present framework, the site lies near 52.0°N, 4.0°E, where Fig. 5 indicates a critical sandpit depth of ~ 10 m, consistent with the reported excavation depths (10–12 m). Although this depth reaches the critical threshold, the pit’s horizontal scale is too small to permit stratification. Its longest axis (~ 1.3 km) is much shorter than the local tidal excursion (~ 6–7 km), resulting in rapid water renewal and short residence times. More importantly, the PUTMOR site is also situated within the Rhine ROFI, where salinity-driven stratification and strong horizontal density gradients influence the water column. Since the present method considers only thermal effects and excludes ROFI-affected waters, its applicability here should be interpreted with care, as it may not be fully valid.

Another offshore example is the Maasvlakte 2 (MV2) harbor expansion, the largest sand extraction in the Dutch North Sea. The northern borrow pit, located ~ 15 km offshore, measures ~ 6 × 2 km and was deepened to ~ 47.5 m, more than 20 m below the surrounding seabed, while the southern pit is smaller (~ 1.6 × 1.2 km) with a depth of ~ 38 m^71^. Dredging in 2009–2013 supplied ~ 220 million m^3^ of sand, demonstrating large-scale extraction feasibility despite uncertain long-term impacts^72^. The MV2 pits, located near PUTMOR, experiences similar tidal conditions and Rhine ROFI influence and is therefore subject to the same limitations of the thermal-only framework. The absence of temperature or stratification measurements makes it a strong candidate for future monitoring.

A comparable case in the German Bight is the Westerland Dredging Area (WDA), the region’s largest sand-mining site located west of Sylt. The WDA covers ~ 10 km^2^, extends ~ 5 km in length and up to ~ 3 km in width, and lies in water depths of 14–34 m^73^. The WDA is located in an area that is already naturally stratified during summer and lies a few kilometers north of the zero-contour of the critical depth in Fig. 5. This implies that the local critical depth is effectively zero, and any additional deepening takes place within a regime where stratification is already present. With sandpit depths of about 12–16 m, the WDA is therefore fully situated inside this stratified zone. Tidal currents in this part of the German Bight are relatively weak, giving a tidal excursion of around 4 km, while the pits are roughly 2 km wide, which would normally reduce the residence time of water inside the excavation. However, because the surrounding water column is already stratified, this residence-time limitation may not apply in the same way here. The area is influenced by freshwater from the Elbe and Weser rivers, with ROFI effects extending > 130 km offshore and introducing salinity-driven density structure and hydrographic complexity. As no measurements of thermal stratification within the WDA pits have been reported, the site represents a valuable candidate for future monitoring of deep excavations in an already stratified, ROFI-influenced water column.

The modeled pit configurations in this study therefore represent realistic extensions of existing operations rather than purely hypothetical cases. By considering pits up to 20 km wide and 20 m deep, we capture scales likely to become relevant as national sand demand grows, illustrating when future borrow areas may begin to affect local currents and water stratification.

### Climate change and future sand demand in the North Sea

Projections of Dutch coastal sea-level rise depend strongly on greenhouse-gas emissions. Under the KNMI’23 scenarios^74^, SSP1-2.6 limits warming to 1.8 °C by 2100, yielding 0.3–0.6 m of rise, whereas SSP5-8.5 implies 4.4 °C warming and ~ 1.2 m by 2100. Beyond 2100, Antarctic ice-sheet instability could raise sea level by 2.5 m by 2100, ~ 5 m by 2200, and up to 17.5 m by 2300^75^, and sea level will continue rising for centuries even if temperatures stabilize^76^.

The Dutch coastal foundation is currently maintained by nourishments of ~ 12 million m^3^ yr^−1^^77^. A strongly simplified estimate based on the ≈ 4000 km^2^ coastal foundation suggests ~ 4 million m^3^ yr^−1^ of nourishment demand per mm of sea-level rise, implying ~ 4 million m^3^ yr^−1^ for 1 mm yr^−1^ and ~ 20 million m^3^ yr^−1^ for 5 mm yr^−1^^78^, although in practice the relationship is not strictly linear. Under the Delta Programme “Warm” and “Steam” scenarios, sea-level rise accelerates from ~ 10 mm yr^−1^ in 2050 to ~ 14 mm yr^−1^ by 2100, requiring 3–4 times present nourishment volumes by mid-century and 4–5 times by 2100. Under high-end IPCC scenarios, the 10 mm yr^−1^ rate is reached around 2065 (RCP 4.5) and 2045 (RCP 8.5), with rates possibly exceeding 60 mm yr^−1^ by 2100. Independent projections indicate ~ 1 m rise by 2100 and up to 3 m by 2200 under RCP 8.5, or ~ 2 m under RCP 4.5^79^, while model studies suggest nourishment demands increase 1.5–3 times for 1 m and 3–6 times for 5 m rise^80^, consistent with^78^. These projections indicate that larger and more offshore sandpits will become increasingly important for coastal protection and climate adaptation. As nourishment demand grows, extraction is likely to shift farther offshore, while additional demand from housing and infrastructure may further increase extraction volumes. In this context, our modeling framework supports the design of sandpits that meet future extraction needs while minimizing impacts. Our results further show that sea-level rise reduces the critical depth for stratification. In the reference case (26 m water depth, tidal velocity 0.47 m s^−1^), the critical depth is about 5 m; assuming constant tidal velocities, this threshold decreases by approximately 20 cm for 1 m sea-level rise and by ~ 70 cm for 5 m rise. Consequently, larger and deeper pits combined with sea-level rise are expected to increase stratification risk at future extraction sites. It should be noted that the critical-depth estimates presented here are based on present thermal forcing and do not account for potential future increases in surface heating. Under warmer conditions, stronger buoyancy input may further promote seasonal stratification and shift the threshold toward smaller excavation depths than those estimated under present climatic conditions.

### Influence of pit shape and orientation

The interaction between tidal flow and a sandpit depends strongly on pit shape and alignment with the main current. Pits aligned parallel to the flow can accelerate currents due to reduced bottom friction, whereas perpendicular pits experience lower velocities because of increased depth^58^. The same study also noted that strong elongation (L > 10 W) is required for internal flow to equilibrate with the ambient current. Similarly, pits with a length-to-width ratio exceeding ~ 4–5 may develop internal velocities that exceed the external flow, depending on acceleration distance and lateral water supply, both controlled by pit geometry^68,81^. For oblique orientations, combined parallel and cross-flow patterns occur. Enhanced velocities were reported for deep pits with small approach angles (< 30°)^58^, while pits rotated by ± 45° showed 2–3 times higher velocities concentrated near pit edges, with weaker flow in the deeper center^68^. Field and laboratory observations support these patterns. At the PUTMOR sandpit^66^, a nearly parallel pit with a length-to-width ratio of ~ 2.5 showed a near-bed velocity reduction of ~ 1/3, consistent with continuity as depth increased from ~ 23 to ~ 35 m. Similarly, laboratory experiments on an irregular, parallel-aligned pit with an aspect ratio of 2.5 reported overall flow reduction^82^. In this study, square pits were used to avoid directional dependence and isolate the influence of pit depth and scale on the hydrodynamic response. This configuration minimizes uncertainties related to alignment and provides a reference case for stratification, capturing the essential flow behavior under orientation-neutral conditions. The results therefore serve as a physical baseline against which the effects of elongation or rotation can be evaluated in future analyses.

### Model limitations and future research

The numerical experiments in this study provide useful insight into sand extraction-induced stratification but involve several simplifications. Firstly, the simulations use idealized, square pits with uniform depth, enabling controlled comparisons but neglecting the irregular geometries, variable depths, and interactions among multiple pits that can modify local flow and mixing in real settings. Secondly, the study only considered thermal stratification and did not include the effects of salinity. This simplification is reasonable for the chosen location, which lies outside the influence of major freshwater inputs such as the Rhine plume. However, in other coastal areas where salinity plays a stronger role or interacts with temperature, additional processes would need to be considered. Including salinity in the stability criterion is conceptually similar to the heating-stirring formulation used here, but more complex^83^. To derive a condition for salinity-driven stratification, the horizontal density gradient is required, which results from the interaction between freshwater buoyancy input and estuarine mixing. The density gradient itself is part of the solution rather than an externally prescribed forcing. Therefore, where density-gradient information exists, the salinity-based formulation can be applied to evaluate the potential for stratification in estuarine environments. It may also serve as a starting point for extending the present framework to estimate the critical sandpit depth in regions influenced by the ROFI. Thirdly, results are averaged over June–July to represent typical summer conditions relevant for ecological processes, which smooths short-term variability such as wind events and spring–neap cycles. In addition, the present simulations do not include wave-driven mixing, as the focus is on summer conditions when thermal stratification is strongest and wave energy is generally lower than during winter. Bed roughness is represented through a roughness length and includes both grain-related roughness and form-related roughness induced by ripples. These roughness components were calculated using a single representative grain size, so spatial variations in sediment grain size and bed composition were not considered. Although these simplifications are unlikely to alter the overall seasonal patterns, but they may affect shorter-term physical or ecological responses. The approach developed here can be expanded in several ways. Applying it to real sand extraction sites with measured seabed shapes and local environmental data would make the results more realistic. Including salinity is planned within another work package of the project that this study is part of, and will help to assess its contribution to stratification, and studying how multiple sandpits interact could improve understanding of cumulative effects under more intensive extraction. Coupling the hydrodynamic model with biogeochemical or oxygen modules, and analyzing the timing and structure of turbulence, would improve understanding of how changes in mixing propagates to ecological responses. Such extensions are especially relevant for plankton dynamics, as phytoplankton production depends on light, turbidity, mixing, and nutrients, and is regulated by zooplankton grazing.

Recent ecological studies in the Southern North Sea have shown the importance of these physical-biological links. Sand extraction, which deepens the seabed and slows near-bed currents, has been shown to affect benthic species differently, favoring opportunistic species such as *Abra alba* while reducing the abundance of more specialized suspension feeders^84^. Combining such ecological insights with the hydrodynamic framework developed here will be essential for understanding how sand extraction affects both physical and biological systems and for guiding ecosystem-based management of future activities in the North Sea.

## Conclusion

This study demonstrates that large-scale sand extraction in the southern North Sea can alter local hydrodynamics and trigger thermal stratification in areas that were previously well mixed. By combining the Simpson–Hunter theoretical framework with high-resolution nested hydrodynamic modeling (GETM-Delft3D), we quantified how excavation depth and horizontal extent jointly affect the balance between tidal mixing and buoyancy-driven stratification. We introduced the concept of a critical sandpit depth, accounting for both local bathymetry and the velocity reduction induced by seabed deepening. Our findings show that excavations in the range of 5–10 m can lead to stratification under specific tidal and depth conditions. Notably, stratification onset depends not only on location and depth but also on pit width relative to tidal excursion length, emphasizing the importance of spatial scale in environmental impact assessments. The results provide a physically grounded screening tool for identifying locations where sand extraction may induce hydrographic changes. From a management perspective, impacts may be reduced by limiting excavation depth in areas where the critical depth for stratification is low, avoiding pit widths comparable to the local tidal excursion length, and selecting extraction sites where natural tidal mixing remains sufficiently strong to prevent persistent stratification. These insights directly support the implementation of the Marine Strategy Framework Directive (MSFD) Descriptor 7, which seeks to prevent permanent alterations to marine hydrodynamics. Beyond the North Sea, the modeling approach and stratification thresholds developed here can support sustainable seabed management in other shelf systems under increasing extraction pressure. Future work should focus on applying this framework to real sandpit geometries, obtaining in-situ temperature observations in sand pits that are likely to approach stratification conditions, incorporating salinity-driven effects, examining how multiple mixing sources such as waves, bed roughness, and short-term atmospheric forcing interact, and coupling hydrodynamic models with biogeochemical modules to assess ecological consequences more comprehensively.

## Supplementary Information

Below is the link to the electronic supplementary material.

Supplementary Material 1

## Data Availability

The datasets generated during and/or analysed during the current study are available from the corresponding author on reasonable request.
