# Supplementary material for: Stratification caused by sand extraction pits in coastal seas
Source: Sci Rep. 2026 May 25;16:23830. doi: 10.1038/s41598-026-48662-5 (PMC13434021; doi:10.1038/s41598-026-48662-5)
Supplement: Supplementary file 1 [file 41598_2026_48662_MOESM1_ESM.pdf]

SUPPLEMENTARY INFORMATION FOR:

Stratification Caused by Sand Extraction Pits in Coastal Seas

Mohammad Daliri[1*] , Johan van der Molen[1]
[1]Department of Coastal Systems, Royal Netherlands Institute for Sea Research (NIOZ), Den Burg, Texel 1790 AB, the Netherlands
mohammad.daliri@nioz.nl, johan.van.der.molen@nioz.nl

ABSTRACT

As demand for marine sand increases across the globe, large-scale seabed excavation in shallow shelf seas raises concerns about its impact on local hydrodynamics and ecosystem functioning. In the Netherlands maritime area of the North Sea, deep sandpits excavated for coastal protection, beach nourishment, and infrastructure projects may reduce tidal mixing and promote thermal stratification, with implications for e.g. oxygen dynamics and biological productivity. This study evaluates how anthropogenic seabed deepening influences stratification onset, focusing on the role of sandpit geometry, including excavation depth and horizontal extent. We combine the energy-based Simpson-Hunter stratification model with a nested hydrodynamic modeling framework, employing GETM for large-scale circulation and Delft3D for high-resolution simulations using 16 sandpit scenarios. We introduce the concept of a critical sandpit depth, defined as the threshold depth beyond which thermal stratification may emerge in summer due to local deepening and associated reductions in tidal current speed. Results from the numerical model confirm that stratification tends to emerge when excavation depth exceeds the local critical threshold derived from the Simpson-Hunter criterion, with deeper and wider pits more likely to trigger stable vertical temperature gradients. In narrower pits, however, stratification is suppressed, likely due to insufficient water residence time relative to the local tidal excursion length. These findings provide a predictive tool for assessing the hydrographic consequences of sand extraction, supporting environmental planning under the Marine Strategy Framework Directive (MSFD). The approach is broadly applicable to other shelf systems for areas with limited salinity gradients, offering operational guidance for identifying excavation limits that prevent stratification and maintaining Good Environmental Status (GES) in marine waters.

Supplementary Figures

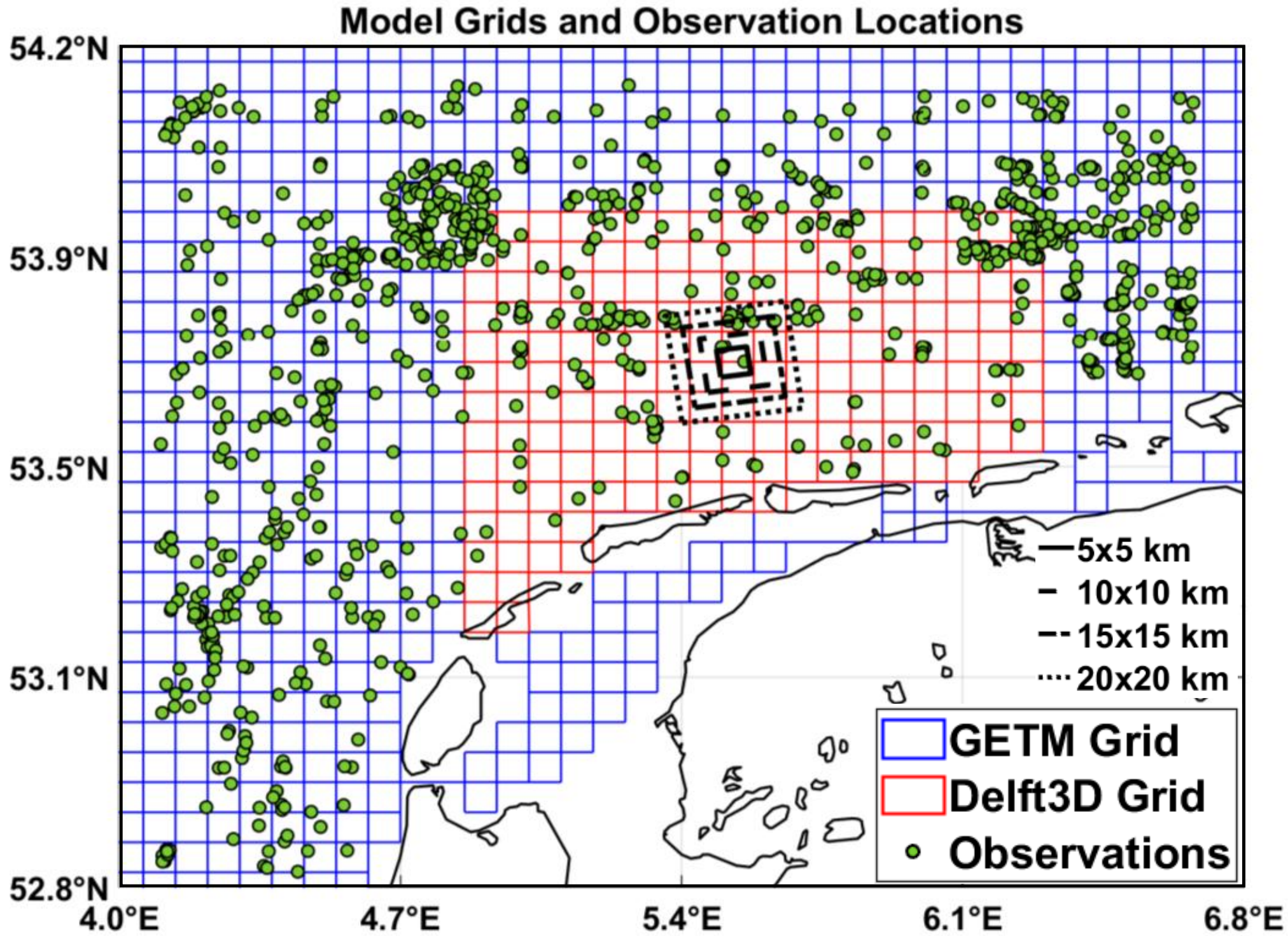

Supplementary Fig. S1. Spatial distribution of model grids and observational points used for validation. The blue grid denotes the GETM model domain, the red grid denotes the Delft3D domain, and black dots indicate ICES observation locations (2017–2019).

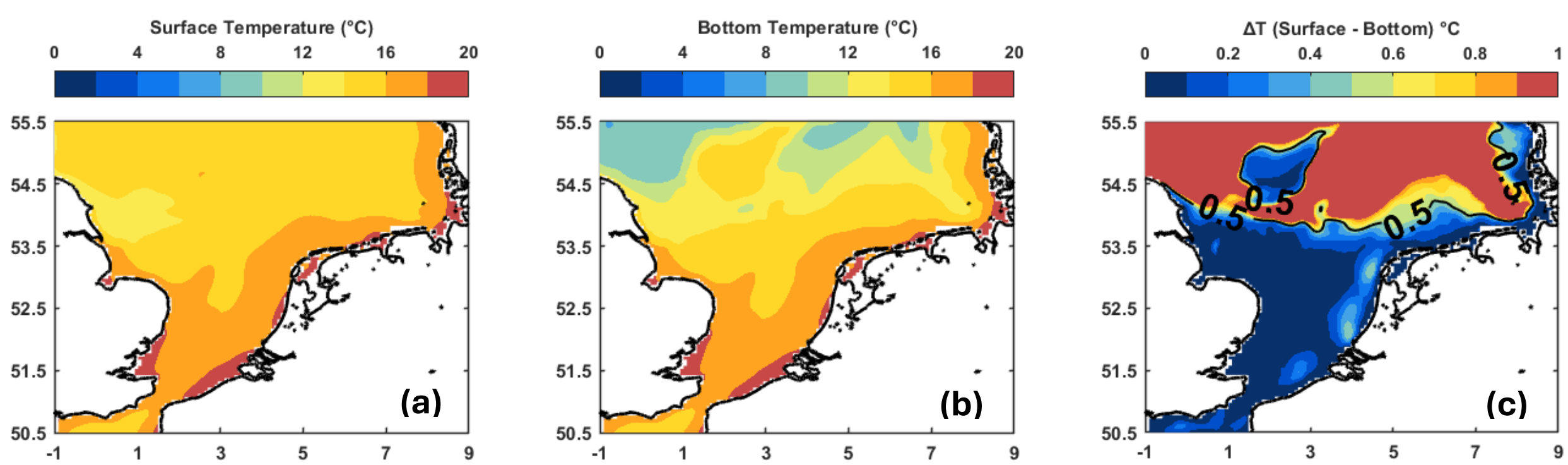

Supplementary Fig. S2. (a) Mean surface temperature, (b) bottom temperature, and (c) vertical temperature difference $\Delta T = T_{surface} - T_{bottom}$ (right) in the Dutch North Sea, averaged over June–July 2019 from GETM output. The black contour in the right panel marks ΔT = 0.5 °C, commonly used to delineate the tidal mixing front.
